# Intestinal Behçet and Crohn’s disease: two sides of the same coin

**DOI:** 10.1186/s12969-017-0162-4

**Published:** 2017-04-20

**Authors:** Simona Valenti, Romina Gallizzi, Dominique De Vivo, Claudio Romano

**Affiliations:** Unit of Pediatrics, Department of Human Pathology in Adulthood and Childhood “G. Barresi”, University of Messina, Viale Consolare Valeria, 98124 Messina, Italy

**Keywords:** Behçet disease, Crohn’s disease, Inflammatory bowel diseases, Intestinal Behçet disease

## Abstract

Behçet’s disease (BD) and Crohn’s disease (CD) are chronic immune-mediated, inflammatory disorders affecting many different systems (joints, skin, eyes, gastrointestinal and biliary tracts). Both disorders have fluctuating courses and when gastrointestinal symptoms are prevalent, differential diagnosis can be difficult. BD involves the gastrointestinal tract in 10–15% of cases with localized lesions in the ileocecal region. The clinical picture is heterogeneous with various clusters of disease expression. CD is a chronic inflammatory disorder, which can affect any part of the intestinal tract, as well as extra-intestinal tissue. Factors that contribute towards the pathogenesis of both disease include the host’s genetic profile, and immune system, and environmental factors such as the gut microbiota. The aim of this manuscript is to provide a narrative review of clinical features of BD and CD, highlighting the importance of differential diagnosis and therapeutic approach, especially in the presence of gastrointestinal involvement. A comprehensive search of published literature using the Pubmed (http://www.ncbi.nlm.nih.gov/pubmed/) database was carried out to identify all articles published in English from 1999 to October 2016, using 4 key terms: “Behçet Disease”, “Intestinal Behçet’s Disease”, “Crohn’s Disease” and” Inflammatory Bowel Disease”.

## Background

Behçet’s disease (BD) was first described by the Turkish dermatologist Hulusi Behçet in 1937 as a syndrome with oral and genital ulcerations and ocular inflammation [1, 2]. It is a rare multi-systemic inflammatory disease with unknown etiology and a chronic recurrent pattern, characterized by recurrent oral and genital aphthous/ulcers with muco-cutaneous, ocular, articular, vascular, and/or gastrointestinal lesions. BD is included both in vasculitis, affecting vessels of all kinds and sizes, and auto-inflammatory disease classifications [3]. Crohn’s disease (CD) is a type of inflammatory bowel disease that may affect any part of the gastrointestinal tract from mouth to anus. It often includes both intestinal (abdominal pain, diarrhoea, vomiting) and extra-intestinal symptoms (fever, weight loss, anaemia, skin rashes, arthritis, inflammation of the eye) [4]. When the gastrointestinal tract is involved, a differential diagnosis between BD and CD is very difficult. There are no pathognomonic laboratory tests or endoscopic findings of intestinal BD, although few, large and deep ulcerations with discrete borders are described as a characteristic endoscopic pattern. Recently, novel diagnostic criteria and a disease activity index have been proposed in the diagnosis of intestinal BD [5]. Treatment for intestinal BD is similar to CD, such as steroids, immunomodulators and biologic agents (anti-tumour necrosis factor α antibody) [6]. The goal of this review is to describe these clinical conditions with similarities and differences from clinical, therapeutic and surgical points of view.

## Epidemiology

BD has the highest incidence in countries located along the ancient Silk Road, stretching from Asia to the Mediterranean countries. It is therefore very common in Turkey (80–370 cases per 100,000 inhabitants), followed by Asia and Middle Eastern countries, including Israel, Saudi Arabia and Iran [7]. Prevalence in the USA and Europe ranges from 0.12 to 7.5 patients per 100,000 inhabitants [8]. Age at onset of BD is usually in young adulthood (25–30 years), but also occasionally in children before the age of 16 years, in 4 to 26% of cases. Intestinal BD occurs in 3–60% of BD patients, with higher frequency of gastrointestinal involvement in East Asian countries such as Korea and Japan than in Western or Middle Eastern countries [1, 9]. Gastrointestinal involvement is higher in patients with childhood-onset [10]. The annual incidence of CD varies from 0 to 20.2 per 100,000 in North America and 0.3 – 12.7 per 100,000 in Europe [11]. It is highest in Western countries in young individuals aged 15 to 29 years [7, 12].

## Pathogenesis

Intestinal BD shares many characteristics with inflammatory bowel disease (IBD), including genetic background, clinical manifestations, and therapeutic strategies. Although etiology is unknown, BD may represent aberrant immune activity triggered by exposure to specific infectious or environmental agents in patients with an underlying genetic predisposition [13]. Human leukocyte antigen (HLA)-B51 is considered the most important genetic factor of BD associated with increased disease severity [14, 15]. The major susceptibility gene for CD is nucleotide oligomerization domain 2/caspase-activation recruitment domain containing protein 15 (NOD2/CARD15) [16]. Caspase recruitment domain-containing protein 9 (CARD9) is a scaffold protein encoded by the CARD9 gene which is located on chromosome 9q34.3. CARD9 belongs to the caspase-associated recruitment domain (CARD) protein family and plays important roles in host defence and immune homeostasis through assembling multifunctional signalling complexes [16]. Mizuki et al., in a genome-wide association study conducted in patients with BD, reported an association for BD with interleukin (IL) 10 and the IL23R-IL12RB2 loci. They identified two suggestive associations on chromosomes 1p31.3 (IL23R-IL12RB2) and 1q32.1 (IL10) both of which predispose individuals to BD [17]. Similarly, IL10 or IL23R variants, although in different polymorphisms, were also observed in IBD patients, suggesting that BD and CD have similar pathogenesis and genetic backgrounds [18, 19]. BD is associated with the intergenic region between IL23R and IL12RB2, while IBD presents an association with variants in IL23R, IL12B, and TYK2 [20]. Increased Th1, Th17, CD4+ and CD8+ T cell, and γδ + T cell activity was found in both the serum and/or inflamed tissues of BD and CD patients, which suggests that innate and adaptive immunity are involved in the pathogenesis of both diseases [18]. Environmental factors also contribute to triggering inflammation, both in BD and IBD, such as smoking, diet, infectious pathogens and antibiotics, medications, lifestyle (stress, sleep and exercise) [11].

## Clinical presentation and diagnosis

**Fig. 1:**
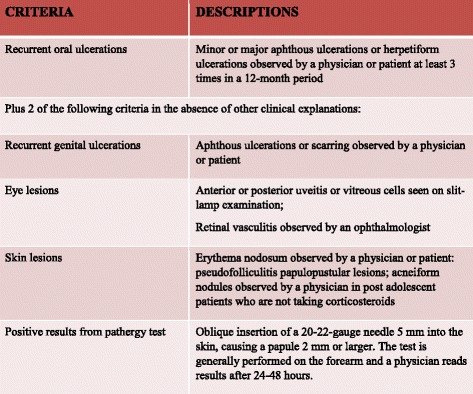
International Study Group Diagnostic Criteria for Behçet’s Disease. Adapted from [30]

**Fig. 2:**
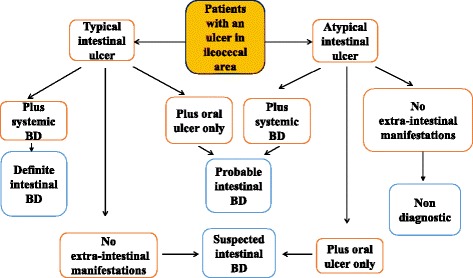
Algorithm for the diagnosis of intestinal BD. Adapted from [5]

**Fig. 3:**
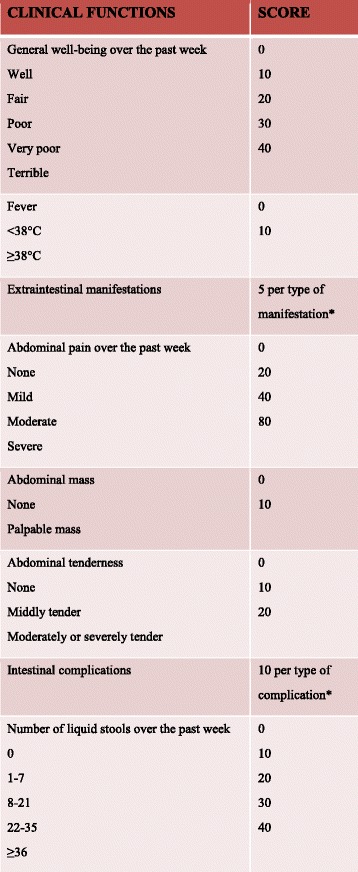
*5 points are added for each type of the following manifestations: oral ulcers, genital ulcers, eye lesions, skin lesions, or arthralgia; 15 points are added for each of the following: vascular involvement or central nervous system involvement. ** Such as a fistula, perforation, abscess or intestinal obstruction. Adapted from [15]

The manifestations of BD are similar to those of IBD, specifically uveitis, arthritis, oral ulcers, pyoderma gangrenosum, vaso-occlusive disease, and thrombotic events [12]. Uveitis in BD can be characterized by chronic pan-uveitis or posterior uveitis with necrotizing retinal vasculitis and tends to be more recurrent and sight threatening than other endogenous uveitis. Other ocular manifestations also include iridocyclitis, keratitis, episcleritis, scleritis, vitritis and optic neuritis [21, 22]. The most commonly reported ocular manifestations in IBD are dry eye, blepharitis, episcleritis, or anterior uveitis. When related to CD, uveitis is frequently bilateral, with insidious onset, and long-lasting, although characteristic acute anterior uveitis with sudden onset may occur [23]. Arthropathy is a common manifestation in BD: arthralgia, oligoarthritis and polyarthritis are the most common reported forms of joint involvement [24]. Arthropathies associated with IBD in the spondyloarthritis group can be divided into axial and peripheral involvement [23]. Genital lesions and neurologic involvement are more common in BD. Vascular complications are present in one third of BD patients. BD involves the gastrointestinal tract in 10–15% of cases with localized lesions that occur in the ileocecal region. Gastrointestinal manifestations usually occur 4.5–6 years later than the onset of oral ulcerations. Sometimes, however, intestinal lesions can precede extra-intestinal manifestations [13]. Anal complications such as stricture, fistula, and abscess formation, which are frequently observed in a third of patients with CD due to its transmural mucosal involvement, are rare in BD, occurring in less than 1% of patients [15, 25]. CD is an inflammatory bowel disease that may affect any part of the gastrointestinal tract from mouth to anus, presenting at onset with a clinical picture characterized by a combination of symptoms/signs such as abdominal pain, diarrhoea, rectal bleeding, nausea, vomiting, abdominal tenderness, weight loss and fever. It often includes, like BD, extra-intestinal signs and symptoms (fever, weight loss, anaemia, skin rashes, arthritis, inflammation of the eye) affecting different systems with skin, ocular, articular lesions [4]. Classically, CD has a clinical manifestation with pain, diarrhoea and weight loss [26]. Similar to adults, children with IBD may present with a range of symptoms, depending on the location, severity and chronicity of inflammation [27]. Children with CD, presenting less specific symptoms than those with ulcerative colitis [28], have a longer period of symptoms prior to diagnosis that contributes to various short- or long-term consequences, including impaired linear growth and delayed pubertal development and inappropriate therapies or interventions [29]. While diagnosis of CD is based on endoscopic and histological features, there is no specific diagnostic test for BD and diagnosis depends on clinical features. In 1990, the International Study Group (ISG) for BD defined the diagnostic criteria of BD. It can be considered in the presence of recurrent oral ulcerations plus 2 of the following criteria: recurrent genital ulcerations, eye lesions, skin lesions, positive results from a pathergy test (Fig. 1) [30]. In 2014, new criteria for BD diagnosis called ICBD (International Criteria for Behcet’s Disease) were proposed and include two additional clinical criteria, neurological and vascular involvement, permitting diagnosis even without the presence of oral aphthous lesions which were considered mandatory in previous ISG classifications [12]. An international expert consensus group (the pediatric BD, PEDBD group) has recently proposed a new set of criteria for the classification of BD in children [31]. These diagnostic criteria for BD do not include intestinal symptoms. Cheon et al. defined novel diagnostic criteria for intestinal BD in Korean patients with ileum-colonic ulcers based on endoscopic features (typical or atypical intestinal BD ulcerations) and clinical patterns (systemic symptoms, oral ulcerations or extra-intestinal manifestations). The positive predictive value and accuracy of these criteria were 86.1 and 91.1% respectively [5] (Fig. 2). Previously, some clinicians had used Crohn’s disease activity index (CDAI) for evaluating intestinal BD activity. CDAI is the most common indicator used in CD relapses and includes some criteria such as: present state of being, abdominal pain, number of bowel movements, haematocrit value, body weight, and administered drugs [32]. The Korean IBD Study Group has developed a disease activity index for intestinal Behçet’s disease (DAIBD) [33]. DAIBD includes clinical features that have been present over the preceding 7 days such as the general condition of patient, extra-intestinal manifestations, intestinal complications, abdominal symptoms and signs, fever and stool frequency not requiring laboratory data or endoscopic findings (Fig. 3). Each item has a single score and total score can differentiate disease activity into “severe,” “moderate,” “mild,” and “quiescent”, showing much higher responsiveness than the CDAI (*r* = 0.812 vs. *r* = 0.645, respectively) but no significant association with endoscopic activity [34]. There are no pathognomonic laboratory tests for BD diagnosis. In the presence of active BD, such as in patients with IBD or other forms or vasculitis, levels of serum markers of inflammation, C-reactive protein and erythrocyte sedimentation rate, are elevated. Anti-Saccharomyces cerevisiae antibodies (ASCA) are anti-glycan antibodies directed against the phosphopeptido mannans found in the cell wall of baker’s and brewer’s yeast (Saccharomyces cerevisiae). Choi et al. have demonstrated that ASCA positivity is possible in up to 44% of patients with intestinal BD and is associated with an increased surgical risk [35]. Antiα-enolase antibodies (AAEA) have been observed in patients with BD. The α-enolase protein is a glycolytic enzyme that serves as a plasminogen receptor on the surface of a variety of hematopoietic, epithelial, and endothelial cells and is crucial in intravascular and pericellular fibrinolytic systems [36]. Evidence suggests that α-enolase plays an important role in autoimmune and inflammatory diseases. Recently, Lee et al. reported that α-enolase is the target antigen recognized by anti-endothelial cell antibodies in the sera of patients with BD. IgM AAEAs were observed in 18 out of 40 patients with BD (45%). Based on their results, they suggest that AAEA has the potential to be a diagnostic marker of BD [36]. Shin et al. assessed the prevalence of IgM AAEA in patients with intestinal BD and found that IgM AAEA can be helpful for the diagnosis of intestinal BD, especially in patients without systemic manifestations of BD. They evaluated the relationships between IgM AAEA and various intestinal BD-related clinical factors, suggesting the association between IgM AAEA and disease activity and severity [37]. Expression of ASCA reflects a specific mucosal immune-mediated response in CD [38]. ASCA frequency in CD patients ranges from 50 to 80% of total IgG and 30 to 50% of total IgA antibodies [39]. ASCA is found more often in CD (50–70%) than healthy controls (<5%); these antibodies increase with age and are associated with a more severe disease course in CD [40]. The sensitivity and specificity of these antibodies in diagnosing Crohn’s disease range from 40 to 70% and 82 to 89%, respectively [41]. In conclusion, for both diseases, clinical diagnosis is not supported by specific serum markers.

## Endoscopic and histological features

**Fig. 4:**
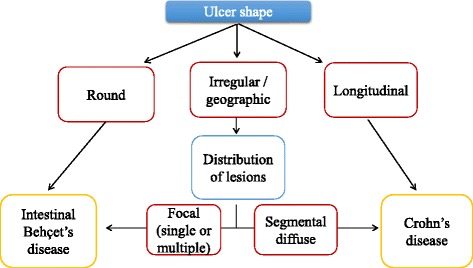
Endoscopic lesions differentiating between intestinal Behçet Disease and Crohn’s Disease. Adapted from [44]

The most frequent localization of intestinal BD, as in CD, is the ileocecal area, although any part of the gastrointestinal tract, including oesophagus, stomach, duodenum, jejunum, colon and extra-intestinal organs, such as liver, pancreas, or spleen, can be affected. Diffuse colonic involvement is rare, but it occurs in approximately 15% of patients who have gastrointestinal involvement [15]. Skipped colonic lesions can be present both in BD and CD [42]. Endoscopic findings of intestinal BD are described as single or few, large, discrete, and round or oval shaped ulcerations in the ileocecal area [43]. However, these lesions vary from small aphthous ulcerations to multiple, irregular shaped ulcerations. In Crohn’s disease, typical endoscopic findings include discontinuous distribution of longitudinal ulcers (defined as ≥4 to 5 cm), cobblestone appearance, and/or small aphthous ulcerations arranged in a longitudinal fashion. Lee et al., comparing colonoscopy findings of 115 intestinal BD and 135 CD patients, have proposed diagnostic criteria [44]. Round shape, fewer numbers (≤5), focal distribution, discrete border, deep penetrating, ileocecal location and absence of aphthous and cobblestone appearance can be suggestive of typical ulcerations of intestinal BD (Fig. 4). There are no pathognomonic histologic findings regarding intestinal BD. There are two forms of intestinal BD lesions: one is mucosal inflammation and ulceration (neutrophilic phlebitis), the other is ischemic damage (vasculitis) [9]. The most common features are: vasculitis affecting small veins and venules; and a normal circumferential mucosa surrounding a large ulceration. Absence of non-caseating granuloma suggests intestinal BD rather than CD, even if non-caseating granulomas are observed in only 15–36% of patients with CD [13]. Histopathological characteristics of CD include discontinuous cryptic architectural abnormalities, discontinuous inflammation, focal cryptitis, and epithelioid granulomas [7] Table 1

**Table 1:** Crohn’s disease and Behçet’s disease: clinical features

	Crohn’s disease	Behcet’s disease
Clinical manifestations	Abdominal pain, diarrhoea, rectal bleeding, nausea, vomiting, weight loss and fever	Oral and genital ulcers, joints and neurological involvement
Extra intestinal manifestations	Uveitis, arthritis, pyoderma gangrenosum, erythema nodosum, iron deficiency anaemia	Uveitis, arthritis, pyoderma gangrenosum, erythema nodosum, vaso-occlusive disease and thrombotic events
Histological features	Discontinuous distribution of longitudinal ulcers, aphthous and cobblestone appearance, focal cryptitis and epithelioid granulomas	Mucosal inflammation and ulceration; signs of vasculitis.
Most involved gender	Female	Male
Genetic predominant factor	NOD2/CARD15 (16p12-q13), CXCL16 (17p13), STAT6 (12q13), TLR4 (9q33), CARD9 (9q34.3)	HLA-B51
Therapy	Systemic corticosteroids 5-ASA/sulfasalazine Thiopurines or AZA/6-MP Anti TNF-α agents Nutritional therapy	Colchicine Systemic corticosteroids Mycophenolate mofetil Cyclophosphamide Thiopurines or AZA/6-MP Anti TNF-α agents
Surgery	Patients refractory to medical treatment or with complications	Refractory to medical treatment or with complications such as perforations, fistulae formation, and massive gastrointestinal bleeding

.

## Therapy

Both BD and CD are multisystem, inflammatory conditions, and steroids with immunomodulatory agents are first-line therapies. The management of patients with BD is based on the presence of organ involvement and disease severity. Colchicine is widely used as first-line treatment for BD (muco-cutaneous and musculoskeletal findings). When colchicine is inadequate and ocular, vascular, neurological, or intestinal involvement is present, steroids and azathioprine can be indicated [7]. A small number of unresponsive patients may require mycophenolate mofetil or cyclophosphamide especially in cases of vascular and neurological involvement [7]. No randomized prospective studies are present on the treatment of intestinal BD, which is very similar to CD. There is controversy regarding the therapeutic effects of 5-amino-salicylates (5-ASA)/sulfasalazine, which have routinely been employed in patients with IBD. 5-ASA should only be used to treat intestinal BD if clinical and endoscopic activity are mild [25]. Systemic corticosteroids (CSs) are often useful as first-line therapy either in the acute phase of intestinal BD or in patients with moderate to severe disease when treatment with 5-ASA/sulfasalazine fails. An initial dose of 0.5–1 mg/kg prednisolone and rapid tapering strategies are prevalent, similar to IBD treatment [13]. A retrospective cohort study in 54 patients with active intestinal BD treated with CSs therapy, showed complete remission in 46.3%, partial remission in 42.6%, and, in 11.1%, no response after a month from treatment. After one year, 35.2% of patients showed corticosteroid dependency [45]. Although CSs are the main treatment for intestinal BD, many patients become CS-resistant or CS-dependent. Thiopurines or azathioprine/6-mercaptopurine (AZA/6-MP) (2.0–2.5 mg/kg/day) are indicated in patients with steroid dependency or resistance. Jung et al., reported cumulative relapse rates of 5.8, 28.7, 43.7, and 51.7% at 1, 2, 3, and 5 years after remission among patients with intestinal BD who received AZA/6-MP [46]. Thalidomide (2 mg/kg/day) has been demonstrated as capable of achieving symptom control and replacing steroid therapy in patients with intestinal BD [15]. Monoclonal antibodies to tumour necrosis factor-α (TNF-α), including infliximab (IFX) and adalimumab (ADA), are biological agents for treating IBD, and are beneficial in patients who are unresponsive to conventional therapies. There are few randomized trials on the use of anti-TNF-α agents in patients with intestinal BD. The first patient with BD treated with infliximab was reported in 2001 [47]. A Korean retrospective multicenter study showed 28 cases of patients with intestinal BD refractory to conventional medical treatment and treated with IFX, with a clinical response rate of 64.8% at 4 weeks [48]. Maintenance infliximab treatment has shown to be more beneficial than short-term treatment for maintaining remission in patients with intestinal BD [15]. Intestinal BD has also been successfully treated with adalimumab, a fully humanized IgG1 monoclonal antibody that binds to TNF-α. The Japanese group in a consensus statement of anti-TNF-α therapy in patients with intestinal BD proposed its indication as a standard therapy for intestinal BD [49]. Recently, adalimumab has successfully been used as a first-line anti-TNF-α agent in patients with steroid-dependent intestinal BD to induce and maintain complete remission [50]. Although there is proven efficacy of anti-TNF-α agents in intestinal BD, further randomized, prospective trials are necessary to confirm these findings. Cantarini et al. [51] has reported efficacy of a novel class of therapies directed against specific cytokines implicated in the disease, as Anakinra. It is an interleukin-1 receptor antagonist with effectiveness in BD with bowel involvement amd resistant to others immunomodulatory agents (anti-TNF-α). Management options for CD include nutritional therapy, drug therapy, and, in severe or chronic active disease, surgery. The aims of CD treatment are to reduce symptoms and maintain or improve quality of life. Steroids are the first-line therapy for CD. The European Crohn’s and Colitis Organization (ECCO) recommend budesonide 9 mg daily to induce remission in mildly active, localized ileocecal CD with 50–60% remission at 8 weeks of therapy [52]. Although it is less efficacious to conventional steroids, especially in the case of severe disease, it has fewer side effects. Moya et al., in a detailed meta-analysis, showed that mesalazine (4 g/day) has a very marginal benefit confirming that budesonide is the best option in mild disease, and found no clear evidence for mesalazine being better than placebo at any dose [53]. Exclusive enteral nutrition therapy is regarded as appropriate only for adjunctive treatment to support nutrition and not for primary therapy, while it is recommended as first line therapy to induce remission in children with active luminal CD [52]. According to ECCO guidelines, moderately active localized ileocaecal CD should be treated with budesonide, or systemic corticosteroids [52]. In the case of steroid-refractory or intolerance, an anti-TNFα based strategy should be used both in adult and pediatric population [54]. In conclusion, medical and surgical therapies are similar in BD and CD but the biological therapy seems to be more efficacious in CD than BD.

## Surgery

When patients with intestinal BD are refractory to medical treatment or present serious complications, such as bowel perforation, severe bleeding, fistulae, obstructions, or abdominal masses, surgical treatment is required. Although remission rates with medical therapy are similar to those reported in CD, in intestinal BD, surgical intervention is more frequent [55]. Intestinal BD requires surgical intervention due to complications such as perforations, fistulae formation, and massive gastrointestinal bleeding, which occur in up to 50% of patients [56]. Park et al. showed that cumulative rates of surgical interventions in intestinal BD are 20% at 1 year, 27–33% at 5 years and 31–46% at 10 years after diagnosis [45]. Many clinical variables have been investigated as predictors of outcomes during medical and surgical therapy: young age, high disease activity at time of diagnosis, “volcano-type” ulcers on endoscopy or colonoscopy, elevated CRP and history of laparotomy confer the poorest prognosis [57]. Surgery is a reasonable alternative for patients with CD refractory to conventional medical treatment and should also be discussed. Surgery is the preferred option in patients with localised ileocecal CD, which requires surgery in 90% of patients with obstructive symptoms [54]. Surgery in CD is not curative: post-operative recurrence rate is lowest when measured by repeat resection, intermediate when clinical indices are used and highest when endoscopy is employed as the diagnostic tool. In population-based studies, the clinical post-operative recurrence rate ranged from 28 to 45% and from 36 to 61% at 5 and 10 years, respectively. It has been demonstrated that the post-operative clinical course of CD is best predicted by the severity of endoscopic lesions [54]. One study compared long-term clinical outcomes between intestinal BD and CD. The probabilities of surgery, hospital admission, and post-operative recurrence were not significantly different between intestinal BD and CD (44.4% vs. 36.0%, 69.2% vs. 73.8%, and 66.5% vs. 79.1% at 10 years, *p* = 0.287, 0.295, and 0.724, respectively), but the rates of corticosteroid and immunosuppressant use were higher in intestinal BD than in Crohn’s disease (59.4% vs. 42.6% and 37.7% vs. 27.1%, *p* < 0.001 and <0.001, respectively) [58].

## Conclusions

Intestinal BD and CD are inflammatory diseases with similar multisystem involvement and various extra-intestinal signs and symptoms. Intestinal BD shares clinical courses, endoscopic and histologic features with IBD, particularly CD. It may be extremely difficult to distinguish intestinal BD from IBD due to similarities in intestinal and extra-intestinal manifestations, and pathologic findings. Differential diagnosis between intestinal BD and CD remains a challenge for clinicians, and both conditions have significant clinical, diagnostic and therapeutic overlaps.

## Abbreviations

5-ASA: 5-amino-salicylates
AAEA: Antiα-enolase antibody
ADA: Adalimumab
ASCA: Anti-Saccharomyces cerevisiae antibodies
AZA/6-MP: Azathioprine/6-mercaptopurine
BD: Behçet disease
CD: Crohn’s disease
CDAI: Crohn’s disease activity index
CSs: Systemic corticosteroids
DAIBD: Disease activity index for intestinal Behçet’s disease
IBD: Inflammatory bowel disease
IFX: Infliximab
IL: Interleukin
ISG: International study group
TNF-α: Tumour necrosis factor-α
